# Bioinformatics analyses of the proteome of *Holothuria tubulosa* coelomic fluid and the first evidence of primary cilium in coelomocyte cells

**DOI:** 10.3389/fimmu.2025.1539751

**Published:** 2025-05-15

**Authors:** Laura La Paglia, Manuela Mauro, Vincenzo Arizza, Alfonso Urso, Sugár Simon, Laszlo Drahos, Vita di Stefano, Claudio Luparello, Mirella Vazzana, Aiti Vizzini

**Affiliations:** ^1^ Institute of High Performance Computing and Networking (ICAR)-CNR, National Research Council of Italy, Palermo, Italy; ^2^ Department of Biological and Technological Science, Chemical and Pharmaceutical Science (STEBICEF), University of Studies of Palermo, Palermo, Italy; ^3^ Mass Spectrometry (MS) Proteomics Research Group, Research Centre for Natural Sciences, Budapest, Hungary

**Keywords:** proteomics, echinoderm, bioinformatics, primary cilium, innate immunity, coelomocytes, coelomic fluid, *Holothuria tubulosa*

## Abstract

The holothurian immune system is characterized by complex defense mechanisms that act through humoral and cellular pathways. Coelomocites are the cellular component of coelomic fluid, and they are involved in host defense, stress response, wound healing, organ regeneration, and tissue homeostasis. The close phylogenetic relationship between *Holothuria tubulosa* and chordate phylum makes it a good model for studying the evolution of immune processes. To elucidate the immune landscape in *H. tubulosa*, we applied an approach combining proteomic analysis of coelomic fluid separated into cellular fraction and extracellular fraction and bioinformatics and in silico analyses. A Search Tool for the Retrieval of Interacting Genes/Protein analysis indicated a highly functional homology to the human protein of immune recognition factors, non-canonical immune-related proteins, signaling molecules, and effector protein, cytoskeleton, and actin remodeling, and provided the first evidence in invertebrate immune cells of an intracellular protein fraction linked to ancestral structure resembling primary cilium involved in cell signaling.

## 1 Introduction

*Holothuria tubulosa* belongs to the phylum Echinodermata, the most ancestral phylum of the deuterostome clade and (1) closely related to chordates. Therefore, it represents an excellent model for studying the evolution of the immune system within deuterostomes. They have a range of highly effective strategies to protect themselves against attack from various pathogens and environmental stresses that arise in response to the fact that they are exposed continuously to potentially pathogenic microorganisms (2–9). The defense activity of sea cucumbers is based on a non-myeloid immune system with specialized cellular and humoral components, which are inside the coelomic fluid. In echinoderms, the coelomic fluid completely fills the coelomic spaces of the body, including the perivisceral coelomic cavities, the water vascular system, and the peripheral systems (10, 11). Coelomocites are the cellular component of coelomic fluid, and they are recognized as biological entities, involved in host defense, stress response, wound healing, organ regeneration, and tissue homeostasis (12). Guatelli et al. proposed that coelomocytes of echinoderms are the functional and morphological homologs of vertebrate blood cells (13), such as thrombocytes, or macrophages, although vertebrate blood cells and coelomocytes differ in morpho-functional complexity and diversity (13).

Coelomocytes of echinoderms include different cell types as follows: phagocytes with roles in graft rejection, chemotaxis, reactive oxygen species production, encapsulation, cytotoxicity, immune gene expression, agglutination, and clotting reactions (14); small lymphocyte cells (4-6 μm), with a large nucleus and a thin layer of cytoplasm whose only common characteristic with their vertebrate namesakes is their morphology, and may be the precursors of other celomocyte types (15); pherule cells (spherulocytes) (15–20), which are characterized by the presence of vesicles in their cytoplasm and some contain pigment (red, yellow, green, brown) with others being colorless. Spherulocytes range in size from 8 to 20 μm and they have been associated with antibacterial activity (21), inflammatory responses, extracellular matrix remodeling (22), and wound healing (23). Crystal cells seem to be exclusive of holothurians. These cells display a very regular geometric morphology (rhomboidal or hexagonal) and present a crystal inclusion within their cytoplasm (17). Their role is still not well defined, but it is likely that they play osmoregulatory roles (15). The vibratile cells range from 6 to 20 μm and are highly motile due to the presence of a flagellum. Their distribution varies according to the species and their function is still not completely understood. They have been associated with clotting reactions (24) and are also thought to be involved in the movement of celomic fluid (15). Queiroz et al. (25) identified coelomocytes of *H. tubulosa* with an integrative approach consisting of living and stained cells, scanning electron microscopy, and morphometric analyses. The results showed seven distinct cell types in these species, including phagocytes, fusiform cells, morula cells, acidophilic spherulocytes, spherulocytes, progenitor cells, crystal cells, and non-identifying vibratile cells (25). The humoral component of coelomic fluid includes different proteins and peptides such as lectins, antimicrobial peptides, lysozyme, enzymes, clotting protein, pattern recognition proteins, Toll receptors, and complement C3 (26).

In humans, primary cilia signaling might modulate specific immune cell phenotypes, behaviors, and functions, which might impact inflammatory responses in the context of syndromes that are termed “ciliopathies” (27). In eukaryotes, primary cilia are polarized structures known for their role as biosensors of shear stress, representing a signaling hub of intracellular and extracellular cues (28). In almost all vertebrate cells, the primary cilia are cellular antennae that receive information from the environment and transmit this information locally into a cellular response (29). Instead, in invertebrates, the presence of primary cilia has been shown in just a few species, such as *Caenorhabditis elegans* and *Drosophila melanogaster*, where they form the basis of several types of sense organs or sensilla and are effectively dendritic extensions of specific neurons (30, 31).

Since many diseases have been connected to cilia that are defective in function, the cilium’s structural performance plays an important role in its function. Recent advances showed that the cilium has a complex structure, containing an array of load-bearing proteins, which regulate its mechano-sensitivity. This shows the importance of cilium mechanics in cellular responses (32). The primary cilium and the better-understood motile cilium and flagellum share very similar basic structures (33). They are all made up of a membrane-bound axoneme that extends upward from the mother centriole/basal body into the extracellular space and contains nine doublet microtubules structured circumferentially. Notwithstanding the shared characteristics, each has unique structural variations that have a significant impact on their mechanics. Indeed, the primary cilium is generally found in different cell types, and it is typically a solitary structure protruding from the cell surface. The typical structural architecture of motile cilia, consisting of nine microtubule doublets together with a central pair, and complemented by dynein arms is not respected; and it lacks the central doublet. The centrosome is directly associated with the development of primary cilia, acting as the organizing hub for microtubules (34). There is evidence of a primary cilium involvement in different signaling pathways that play crucial roles in various cellular processes, such as differentiation, cell cycle, tissue homeostasis, and the immune response. In particular, the primary cilium has been associated with the Hedgehog (Hh) and Wintless (Wnt) signaling pathways, which are essential for the maintenance of tissue, cellular homeostasis, and immune respose. Wnt signaling is a primary regulator of cell polarity, cell development, and the preservation of cellular homeostasis (34). In humans, the first direct connection between primary cilia and immune cells seems to occur via the centrosome. The centrosome constitutes a hybrid organelle, which serves as a plasma membrane-associated primary cilium organizer and a juxtanuclear microtubule-organizing center (35). In this study, we used a proteomic approach to study the proteins and signaling pathways involved in the different roles of caelomocites in the holothurian *H. tubulosa.*

Furthermore, bioinformatics analyses suggest the presence in *H. tubulosa* of a putative structure resembling human primary cilium, which may be conserved through evolution.

## 2 Materials and methods

### 2.1 Animals

In total, 60 healthy adult specimens of *H. tubulosa* (length 11 ± 0.98 cm and body weight 46 ± 7.5 g) were collected in the Gulf of Palermo (Sicily, Italy). The animals were acclimatized for 1 week in the laboratory tanks in a constantly aerated aquarium at a temperature of 15 ± 2°C and were fed with a commercial invertebrate feed until 24h before the sampling (Algamac 3000, Aquafauna BioMarine Inc., Hawthorne, CA, USA).

### 2.2 Sample preparation

The coelomic fluid (CF) was collected by making a 3–5 cm incision on the antero-dorsal side of each individual using a scalpel, allowing the fluid, composed of both humoral and cellular components, to percolate naturally into 50 mL Falcon tubes pre-filled with Etylenedimine tetra-acetic acid (ISO-EDTA) anticoagulant (20 mM Tris, 0.5 mM NaCl, 70 mM EDTA; pH 7.5). This procedure ensured the prevention of clotting and the preservation of cellular integrity. The CF was collected, kept on ice, and immediately centrifuged at 1,000 g for 10 minutes at 4°C to separate the cellular component (cell pellet) and the humoral component (cell-free coelomic fluid). The cell pellet was pottered and sonicated with 1X RIPA buffer added with 1:200 antiprotease. The sample was centrifuged at 20,000 g for 10 min at 4°C. The supernatant (cell pellet lysate) and the cell-free coelomic fluid were frozen at -80°C and lyophilized. To obtain a sufficient amount of proteins for the analysis, all cell lysates were combined to form a single sample. The same procedure was followed for the cell-free coelomic fluids. The proteins presented in the humoral fraction correspond to extracellular proteins that have been secreted by the coelomocytes and the cellular component refers to the intracellular proteins that have been isolated from the coelomocytes.

### 2.3 Proteome

The freeze-dried samples were dissolved in 0.25% RapiGest (Waters Co., Milford, MA, USA) and passed through Microcon 10 kDa filters (Merck KGaA, Darmstadt, Germany) to remove the RIPA buffer (the flowthrough was discarded). The nominal protein concentration was then measured for each sample using a NanoDrop 2000 UV-VIS spectrophotometer (Thermo Fisher Scientific, Waltham, MA, USA). Moreover, 10 µg aliquots of each sample were taken in 30 µl of 5% methanol, reduced using RapiGest and dithiothreitol for 30 minutes at 60°C (Thermo Fisher Scientific, Waltham, MA, USA), and subsequently alkylated using iodoacetic acid (Thermo Fisher Scientific) for 30 minutes at room temperature in the dark in 25 mM ammonium bicarbonate buffer (Thermo Fisher Scientific). Samples were digested in-solution using endoproteinase LysC-trypsin (Mass Spec grade, Promega, Madison, WI, USA) at a 1:100 ratio for 1 h and then trypsin (Mass Spec grade, Promega) at a 1:25 ratio for 3 h at 37°C. Proteolysis was stopped by adding 1 µl of 100% formic acid (Thermo Fisher Scientific), and the samples were dried down and cleaned up using C18 spin columns (Thermo Fisher Scientific) according to the manufacturer’s protocol. The cleaned peptide extracts were dried down and stored at -20°C until further analysis. Aliquots of a nominal 1 µg of the tryptic digests were analyzed using a Dionex Ultimate 3000 nanoRSLC (Dionex, Sunnyvale, CA, USA) coupled to a Bruker Maxis II ETD mass spectrometer (Bruker Daltonics GmbH, Bremen, Germany) via a CaptiveSpray nanobooster ion source. The samples were first loaded on the trap column using 0.1% trifluoroacetic acid at a flow rate of 5 µl/min for 8 minutes using an Acclaim PepMap100 C-18 trap column (100 µm × 20 mm, Thermo Scientific). For the gradient elution, an ACQUITY UPLC M-Class Peptide BEH C18 column was used (130 Å, 1.7 µm, 75 µm × 250 mm, Waters) at a 300 nL/min flow rate and 48°C column temperature using a linear gradient from 4% B to 50% B in 120 minutes. Solvent A was 0.1% formic acid, and solvent B was acetonitrile with 0.1% formic acid. The cycle time for data-dependent acquisition was 2.5 s. MS spectra were acquired at 3 Hz, while MS/MS spectra were acquired at 4 or 16 Hz, depending on the intensity of the precursor ion. Singly charged ions were excluded from the analysis (36). The protein quantification was based on the mass-to-charge ratio, which represents the ratio of the mass number and the charge number, *z*.

**Figure 1:**
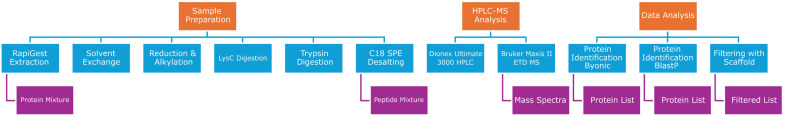
Workflow of proteomic data analysis: orange squares show the main steps of proteomic data workflow, which are sample preparation, HPLC-MS analysis, and computational data analysis. Each of the three main steps requires different passages (blue squares). Finally, the purple squares indicate data output sorting from the different steps of the proteomic workflow.

The proteins were first identified by searching against the Uniprot Aechinodermata (downloaded: 07/03/2020) database using the Byonic software search engine (v3.8.13, Protein Metrics Inc, San Carlos, CA, USA) (RRID: SCR_016735) with the following parameters: 1% false discovery rate (FDR), 20 ppm peptide mass tolerance, 30 ppm fragment mass tolerance, two missed cleavages, trypsin as the enzyme, carbamidomethylation of cysteines as the fixed modification; and the following variable modifications: Oxidation/+15.994915 @ M, Deamidated/+0.984016 @ N, Deamidated/+0.984016 @ Q, Gln- >pyro-Glu/-17.026549 @ NTerm Q, Glu->pyro-Glu/-18.010565 @ NTerm E. Protein hits were filtered using Scaffold (version 4.11, Proteome Software, Inc., USA) (RRID: SCR_014345) using the same parameters stated above in addition to the following parameters: protein grouping strategy, Experiment-wide grouping with protein cluster analysis; peptide thresholds, 95.0% minimum; protein thresholds, 1% FDR and 2 peptides minimum. Subsequently, protein identification was also performed using BlastP comparison to non-redundant protein sequence and model organism databases (available at https://blast.ncbi.nlm.nih.gov/Blast.cgi?PAGE=Proteins; accessed in November 2024) (RRID: SCR_004870). An expected value of < 1 was set as cutoff. **Figure 1** shows the workflow of proteomic data, starting from sample preparation to downstream data analysis (**Figure 1**).

### 2.4 Protein annotation

The Uniprot web tool (https://www.uniprot.org/) (RRID: SCR_002380) was used for protein annotation. It is a web resource for protein sequence and functional information through ID mapping (https://www.uniprot.org/id-mapping).

### 2.5 String analysis

The Search Tool for the Retrieval of Interacting Genes/Protein (STRING) database (RRID: SCR_005223) (https://string-db.org/), which allows the visualization of complex networks (through clustering analysis), was used to retrieve the predicted interactions for the identified proteins. This web tool returns different information: first, it can reconstruct potential protein-protein interactions, aiming to evidence specific sub-networks from input data. Second, the web tool can perform clustering analysis. Finally, STRING can provide information on functional enrichment and pathway analysis for each cluster. We used the following settings for STRING analysis: full STRING network as the network type, in which edges indicate both functional and physical protein associations; evidence as the meaning of the network edges; different types of interaction sources, such as text-mining, neighborhood, experiments, co-occurrence, and more; and a medium confidence cut-off of 0.400 as the minimum required interaction score to highlight the more significant interactions. Proteins were clustered according to the k-means algorithm, an unsupervised clustering algorithm based on an adjacency matrix, and values with a cut-off score of FDR <0.05 were considered statistically significant and evaluated for further analysis. Each cluster produced by the algorithm was analyzed using Gene Ontology (GO). The STRING database is also linked to the SMART database (http://smart.embl-heidelberg.de/) (RRID: SCR_005026), which allowed us to compare different protein domains of proteome data of *H. tubulosa* and compare them with domains present in the human species.

Besides the STRING “clustering” function, the “homology search” function (string- db.org/cgi/proteinhomology) was used to calculate the percentage of protein sequence similarity between different species. *H. tubulosa* extracellular protein fractions were compared with those of the *Homo sapiens* species, and protein sequences were blasted and evaluated by using a STRING similarity bit score value. These are Smith–Waterman scores calculated for alignments between two random or unrelated sequences and are internally used by STRING as a proxy for protein homology. The bit score measures sequence similarity independent of query sequence length and database size and is normalized based on the raw-pairwise alignment score. The original scores were computed by the similarity matrix of proteins (SIMAP) project (SIMAP, http://mips.gsf.de/simap/) (RRID: SCR_007927). The bitscore is defined by “*S*” and it is determined by the following formula:


S=(λ×S−lnK)/ln2

Where:

*λ* is the Gumble distribution constant,

*S* is the raw alignment score,

*K* is a constant associated with the scoring matrix used.

Clearly, the bit score (*S*) is linearly related to the raw alignment score (*S*). Thus, the higher the bit score, the more highly significant the match is. The bit score provides a constant statistical indicator for searching different databases of different sizes or for searching the same database at different times as the database grows (www.biostars.org/p/187230/). The “homology search” mode is the most popular strategy for inferring functional similarity between the same proteins of two different species. It is known that homologous sequences have similar structures, and frequently, they have similar functions as well. Scores less than 50 were not significant, and proteins with scores less than 50 were discarded from further analyses.

## 3 Results

### 3.1 Proteomic analysis of extracellular fraction

Proteomic analysis of extracellular fractions produced 174 unique annotated proteins. A workflow of proteomic data analysis is shown in **Figure 1** where detailed steps of sample preparation and data analysis are summarized (see **Figure 1** in the Materials and Methods section). The Uniprot web tool was used for protein annotation analysis (https://www.uniprot.org/).

Of the 174 proteins identified by mass spectrometry, 6 were common contaminants and were discarded from subsequent analysis, and 57 proteins were annotated as uncharacterized proteins. This means that, although they have been identified experimentally, they still do not have a known functional or structural domain. Moreover, these proteins do not have a homolog in humans, and they were also discarded from the analysis. Furthermore, 27 proteins did not have information about coverage percentage or log probability score from MS analysis, and thus 111 annotated proteins remained that were suitable for the subsequent analysis. They were analyzed using the STRING web tool to elucidate the potential interactions between the proteins found by the humoral fraction. When analyzing the remaining 111 proteins in the STRING database, the tool returned as the output of the analysis just 59 proteins, thus the remaining portion was lost during this last analysis.

**Figure 2:**
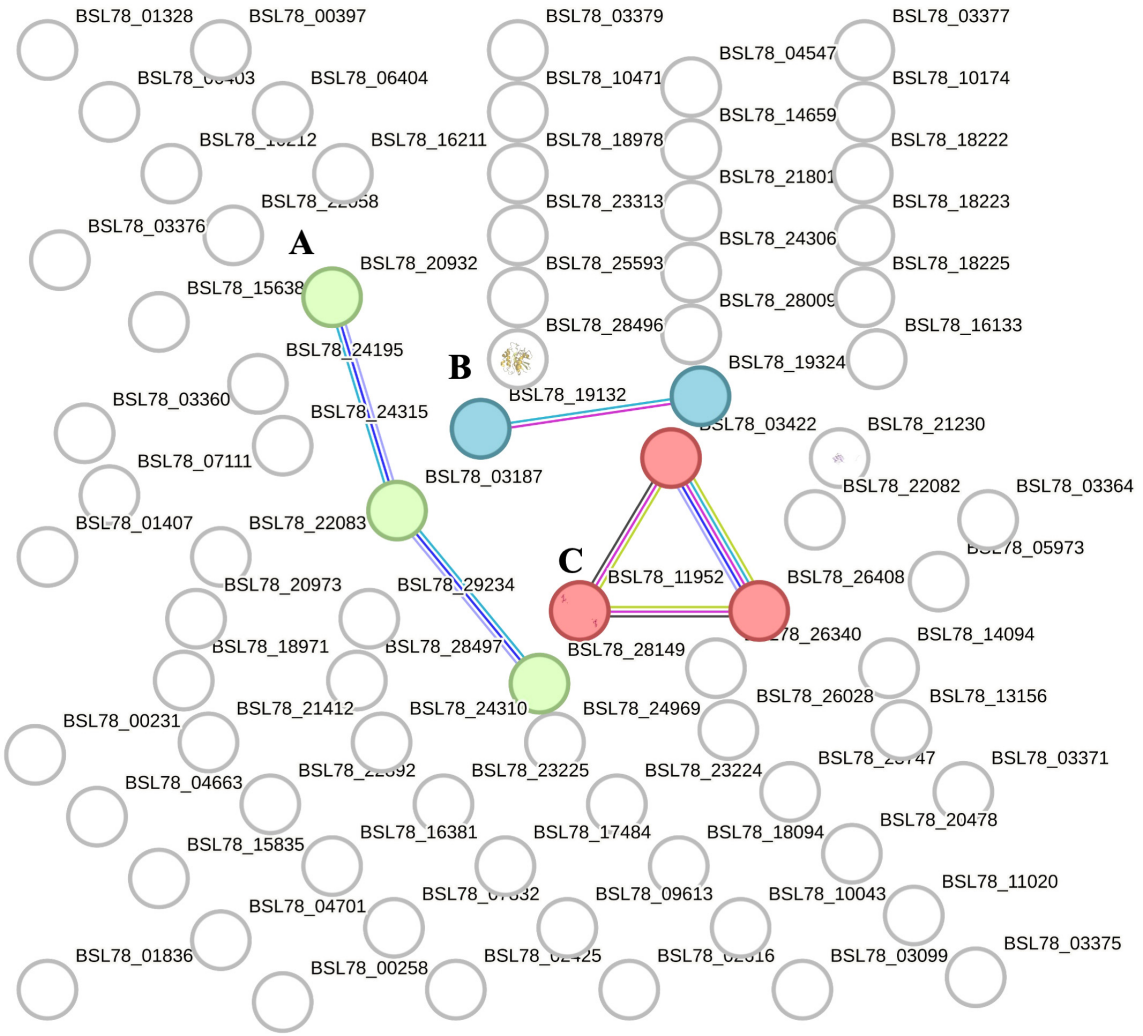
STRING analysis of the extracellular protein fraction. Capital letters **(A–C)** indicate the different clusters evidenced by the STRING analysis. Cluster **(A)** indicates proteins belonging to the fibrinogen family. Cluster **(B)** is composed of Vitellogenin domain-containing protein (VTG domain-containing protein) and putative endoplasmic reticulum resident protein 44 (ERp44). Cluster **(C)** consists of Histone proteins H1-βbeta, H2A, and H2B.

Three main clusters (**Figure 2**, clusters A–C) were found, and they were composed as follows: the first cluster was composed of a few proteins linked to fibrinogen (cluster A, three proteins), the second cluster (cluster B, two proteins) contained Vitellogenin domain-containing protein (VTG domain-containing protein) and a putative endoplasmic reticulum resident protein 44 (ERp44), and the third cluster (cluster C, three proteins) consisted of Histone proteins H1-β, H2A, and H2B. Besides the STRING “clustering” function, we used the “homology search” (string- db.org/cgi/proteinhomology) function that calculates the percentage of protein sequence similarity between different species. Thus, the sequences of *H. tubulosa* extracellular protein fractions were compared with *H. sapiens*, and protein sequences were blasted and evaluated using a STRING similarity bit score value.

**Table 1** shows the annotated proteins of the humoral fraction of coelomic fluid and their relative STRING similarity sequence analyses. Sequence homology analysis of extracellular protein fraction showed a high sequence conservation between *H. tubulosa* and humans. Indeed, the STRING similarity scores were between 639.4 (DMBT1), the highest homology score, and 73.2 (FREP-A). Most immune molecules showed a similarity score of approximately 150, indicating a high protein sequence similarity. Functional analysis of proteins in the humoral fraction of coelomic fluid allowed us to classify them into four main categories: immune receptor factors (IRFs), non-canonical immune proteins (NCIPs), signaling molecules, and effector proteins.

**Table 1:** Immunity-related proteins of an extracellular fraction of coelomic fluid, grouped by function: immune recognition factors (IRFs), non-canonical Immune-related proteins (NCIPs), signaling molecules, and effector proteins.

Immune-related proteins				
Pattern recognition receptors: Immune recognition factors (IRFs)				
Cellular function/pathway/category	Protein ID	Protein name	Human homolog (Gene ID)	STRING similarity bit score
SCRC	A0A2G8K857	Putative deleted in malignant brain tumors 1 protein (DMBT1)	DMBT1	639.4
Lectin	A0A2G8LE58	Putative techylectin-5B-like	ANGPTL1	124.4
Pattern recognition receptors (PPRs)/Innate defense protein with bacteria binding domains	A0A2G8KAA3 A0A2G8JT06 A0A2G8KAD0 A0A2G8JX06 A0A2G8KYJ1 A0A2G8JM67 A0A2G8L6S5	Putative ficolin-2-like proteins (FLN)	FCN1 TNR FCN2 ANGPTL2 FCN2 FIBCD1 FCN1	135.6 114.4 170.0 107.1 124.0 141.7 158.7
Fibrinogen-related proteins (FREPs)/putative pattern recognition receptor	A0A2G8K011	Fibrinogen-like protein A	TNR	147.5
	A0A2G8JGZ6 A0A2G8JHE8 A0A2G8LA71 A0A2G8JP71 A0A2G8LDR8 A0A2G8K2K9 A0A2G8LHK6 A0A2G8LHG3 A0A2G8LHJ1 A0A2G8LHI9 A0A2G8KFX9	Fibrinogen-like protein	FIBCD1 FCN2 ANGPTL1 FCN2 TNR FCN2 FCN1 FCN1 ANGPTL2 TNR ANGPTL2	178.7 162.9 82.0 174.5 190.7 201.1 120.2 125.6 149.1 128.6 142.5
	P19477 0A2G8LNH1	Fibrinogen-like protein A (FREP-A)	FCN1 TNR	95.1 73.2
	A0A2G8KXG0	Putative fibrinogen-like protein A-like	FCN1	124.4
	A0A2G8JW85 A0A2G8LI29 A0A2G8JSV5 A0A2G8LI63 A0A2G8KKG9 A0A2G8K3T5 A0A2G8JVZ9 A0A2G8KHJ3 A0A2G8KA74 A0A2G8K2I6 A0A2G8KGA5 A0A2G8LHK5	Fibrinogen C domain-containing protein 1 (FIBCD1)	ANGPTL1 FCN1 TNR FCN2 FIBCD1 FCN1 FCN1 FIBCD1 TNR ANGPTL1 TNR TNR	117.1 167.9 156.8 179.5 190.3 99.0 170.6 142.9 182.6 141.0 137.1 148.7
	A0A2G8KGA5	Tenascin (TN)	FCN1	95.0
Antibacterial activity/NET	A0A2G8LHK5	Histone H1- β	H1-5	95.1
	A0A2G8KT62	Histone H2A	H2AX	223.4
	P48557	Histone H2B	H2BU1	194.5
Pattern recognition receptors: non-canonical proteins interacting with pathogens (NCIPs)				
	A0A0E3VJX4	Beta-actin	ACTG1	362.5
	V5YU14	Collagen	C1ǪTNF2	79.9
Signaling				
Vesicular trafficking/	A0A286T421	Calmodulin (CaM)	CALM2	279.3
	A0A2G8K1M3	ADP-ribosylation factor family (Arl)	ARF1	337.4
	A0A2G8LLR8	Putative TBC1 domain family member 17	TBC1D15	165.5
actin remodeling/Ca2+binding	A0A2G8K739	Putative ER resident protein 44 (ERp44)	ERP44	330.9
	A0A2G8KH34	Put. huntingtin-interact. protein1 isoform X3 (HIP-1)	HIP1	247.2
	A0A2G8LR54	GTP 3’,8-cyclase	MOCS1	483.8
Polyubiquitination	A0A2G8LRI2	Putative E3 ubiquitin-protein ligase (HUWE1)	HUWE1	1862.8
Cell polarity/cytoskeleton	A0A2G8KLY6	Put. protocadherin-like wing polarity protein stan	PCDHGA12	110.2
	A0A2G8K162	Put. dynein heavy chain 1, axonemal (DYNC1H1)	DNAH1	262.9
	P35527	Keratin, type I cytoskeletal 9 (KRT9)	KRT9	689.5
ECM	A0A2G8KZU0	Putative angiopoietin-2-like (Angpt-2-like)	FCN3	77.0
	A0A2G8KAK2	Angiopoietin-like 1 (Angpt-1)	ANGPTL1	166.0
	A0A2G8L4Ǫ2	Put. inter-alpha trypsin inhib. heavy chain H3 (ITIH3)	ITIH4	86.7
	A0A2G8K7M8	Vitellog. domain-contain. Prot. (VTG contain. prot.)	APOB	234.6
Effector proteins				
Transferrin-family proteins	A0A0K1Z4Z0	Melanotransferrin 4 (Mtf4))	MELTF	140.2
	C4TǪH7	Major yolk protein 1 (MYP-1)	MELTF	126.3
	C4TǪH8	Major yolk protein 2 (MYP-2)	MELTF	114.0

As shown in **Table 1**, the first class of immune-related proteins can be divided into different sub-classes such as scavenger receptor cysteine-rich (SRCR) proteins [Putative deleted in malignant brain tumors 1 protein (DMBT1)], lectins (putative techylectin-5B-like), pattern recognition receptors (PPRs) (putative ficolin-2-like protein), fibrinogen-like family (FREP) proteins, and molecules with antibacterial activity or involved in neutrophil extracellular trap (NET) formation. The second class identified in the humoral component of coelomic fluid is represented by non-canonical proteins interacting with pathogens (NCIPs). To this second class belong proteins such as beta-actin and collagen. The third class of immunity-related proteins is involved in the signaling cascade. It includes vesicular trafficking molecules, calcium-binding proteins, cell polarity and cytoskeleton proteins, extracellular matrix (ECM) proteins, and finally polyubiquitination proteins. The fourth class of immune-related proteins found in the humoral compartment is represented by effector proteins. They belong only to the transferrin family: melanotransferrin-4 (MELTF-4) and Major York proteins 1 and 2.

### 3.2 Proteomic analysis of intracellular fractions

**Figure 3:**
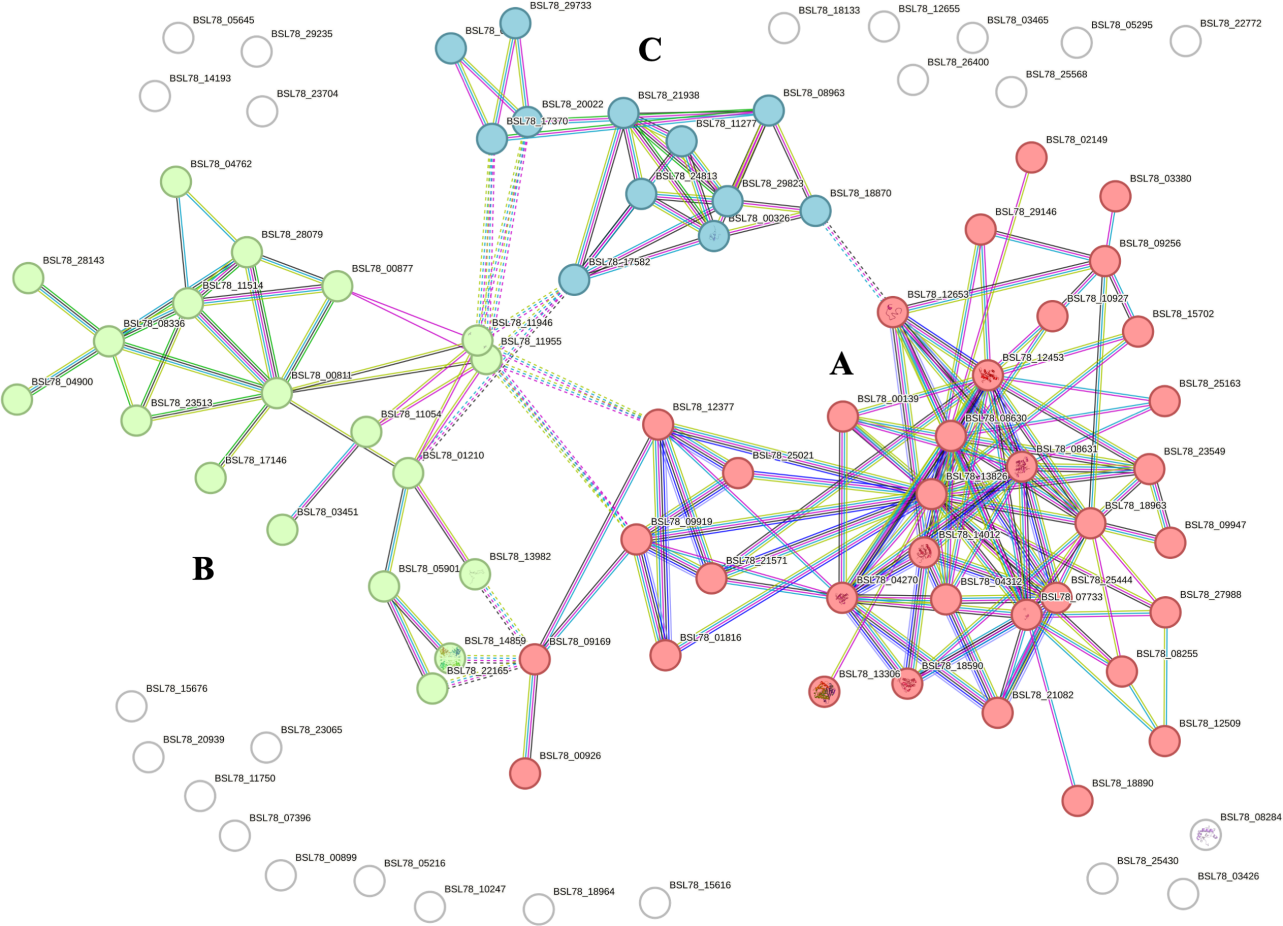
STRING analysis of the intracellular protein fraction. Capital letters **(A–C)** indicate the different clusters evidenced by the STRING analysis. Cluster **(A)** is represented by proteins linked to cell signaling, cytoskeleton, and actin remodeling. Cluster **(B)** includes proteins of various metabolic pathways. Cluster **(C)** is related to ribosomal activity, primary cilium, and UPS machinery.

**Figure 4:**
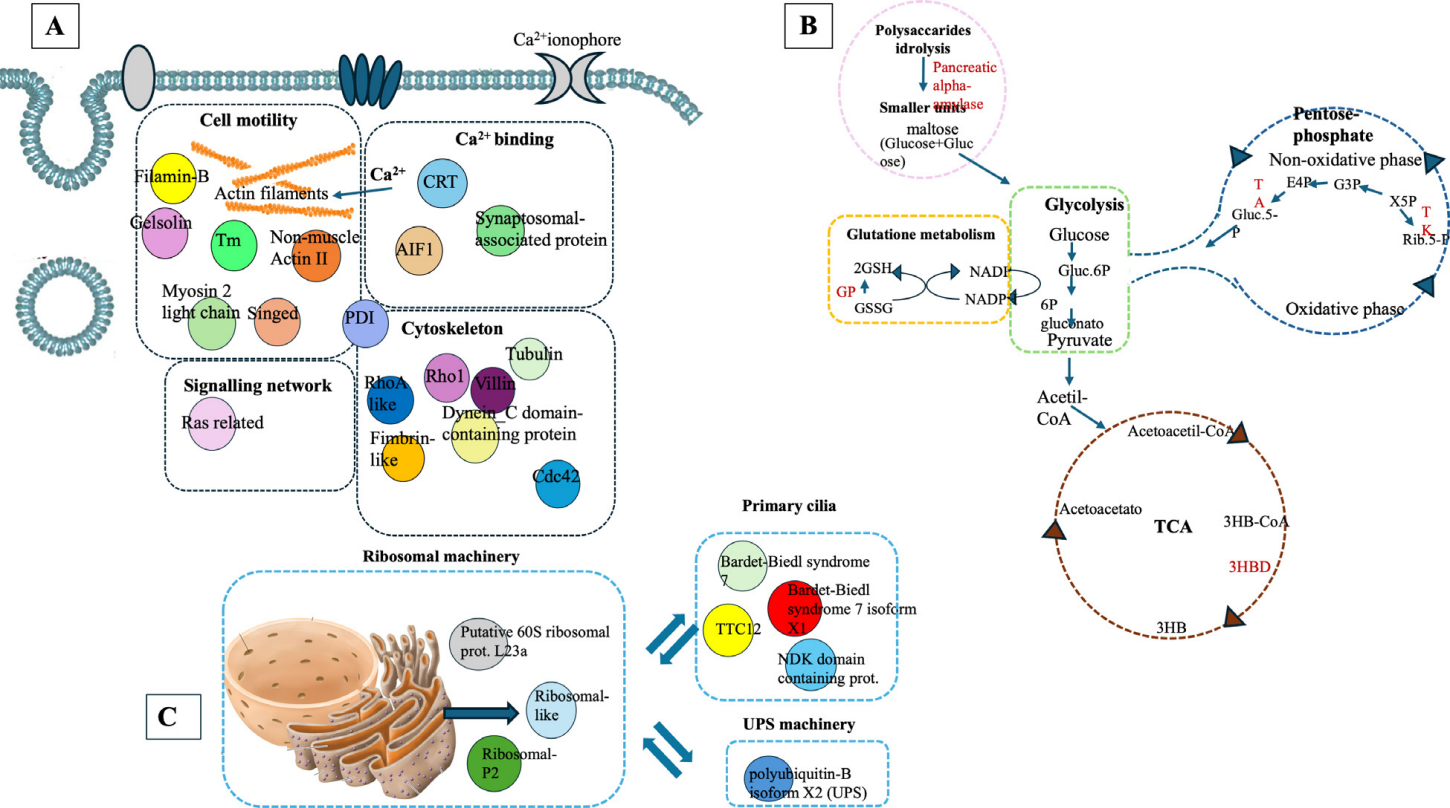
Graphical representation of proteins linked to Cluster **(A)**, Cluster **(B)**, and Cluster **(C)** in the intracellular component of coelomic fluid.

Proteomic analysis of intracellular fractions produced 215 annotated proteins. STRING analysis of the intracellular fractions of *H. tubulosa* coelomic fluid proteins evidenced three different clusters (**Figure 3**). Cluster A was mainly represented by proteins linked to cell signaling, cytoskeleton, and actin remodeling. Cluster B included proteins of different metabolic pathways. Cluster C was linked to ribosomal activity, ubiquitin proteasome system (UPS) machinery, and primary cilium. A graphical representation of some of the proteins present in the three clusters is also shown in **Figure 4**.

**Figure 5:**
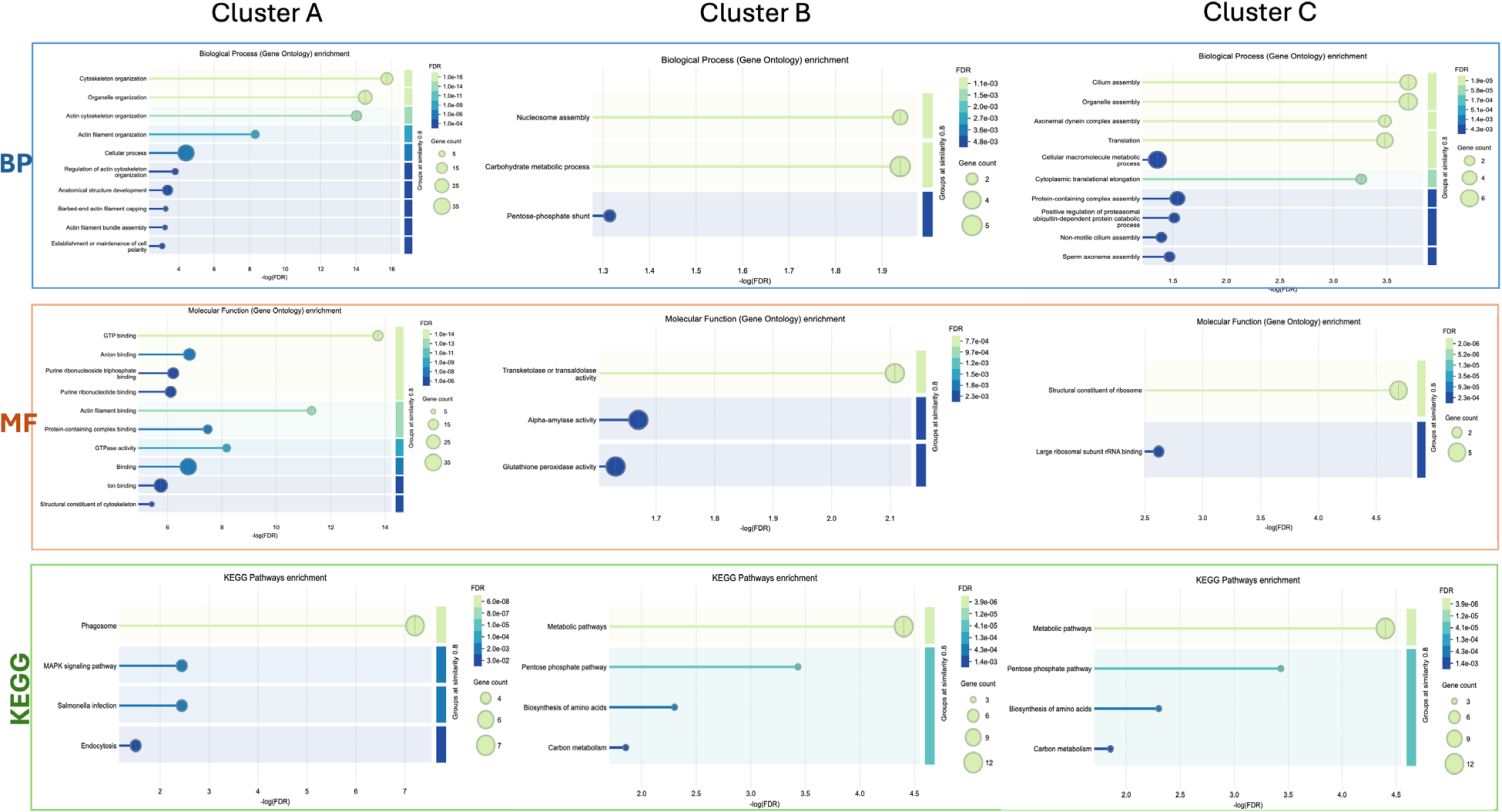
Graphical representation of STRING Gene Ontology (GO) analysis of the GO terms biological processes (BP) and molecular function (MF), and KEGG pathways for intracellular protein Clusters **(A, B)**, and **(C)**.

Cluster A (**Figure 3**) refers to proteins linked to different cellular mechanisms such as cell motility, calcium binding, cytoskeleton remodeling, or signaling networks. STRING analysis found GO terms linked to biological processes (BP) such as synaptic vesicle priming and actin filament network. Furthermore, molecular function (MF) and cellular component (CC) terms confirmed BP ontology, with MF terms such as actin filament binding, structural constituent of the cytoskeleton, and phosphatidylinositol-4,5-biphosphate binding, and CC terms such as actin and cytoskeleton (**Figure 5**). This last cluster is mainly represented by proteins belonging to pathways such as phagosome, Salmonella infection, MAPK signaling, and endocytosis, showing the putative role of these proteins in processes linked to vesicle trafficking and cytoskeleton remodeling after pathogen stimuli (**Figure 5**). A detailed list of proteins linked to GO and Kyoto Encyclopedia of Genes and Genomes (KEGG) pathway STRING analysis is in the **Supplementary Material** (**Supplementary Material Table**, sheet GO and KEGG cluster A, sheet proteins A). A STRING homology search was also applied for all the proteins belonging to cluster A to evidence the sequence homology between *H.tubulosa* and *H. sapiens*. The results of STRING homology search are shown in **Table 2**. The STRING similarity scores were between 944.1 (FLNA), which is the highest homology score, and 84.3 (PLS3). Most molecules showed a similarity score of approximately 300, indicating a high sequence similarity.

**Table 2:** STRING homology analysis of cluster A proteins.

Protein ID	Protein name	Human homolog (Gene ID)	STRING similarity bit score
A0A2G8LRǪ1	Synaptosomal-associated protein	SNAP-25	232.3
A0A2G8LPL8	Putative calreticulin-like isoform X2.	CALR	117.5
A0A2G8LLX1	Putative tubulin alpha chain.	TUBA1A	302.4
A0A2G8LL48	Villin.	VIL-1	471.9
A0A2G8LHI5	Techylectin-5B	TNR	118.2
A0A2G8LEX4	Putative ras-related protein.	RAB7A	342.4
A0A2G8LF11	Synaptosomal-associated protein	SNAP-25	232.3
A0A2G8L522	F-actin-capping protein subunit beta	CAPZB	450.7
A0A2G8L3I6	Putative fimbrin-like.	PLS3	84.3
A0A2G8L2K9	Rho1.	RHOA	323.6
A0A2G8L2L6	Putative transforming protein RhoA-like.	RHOA	344.0
A0A0H4BK46	Protein disulfide-isomerase, PDI.	P4HB	523.9
A0A2G8L0V3	Putative alphaP integrin isoform X1	ITGA8	392.1
A0A2G8KYX9	Tubulin beta chain.	TUBB4B	914.8
A0A2G8KYU0	Allograft inflammatory factor 1	AIF-1	122.9
A0A2G8KWA4	Putative filamin-B	FLNA	944.1
A0A2G8KRX3	Tubulin beta chain	TUBB4B	918.7
A0A2G8KRP0	Cdc42	CDC42	287.3
A0A2G8KRL6	Protein singed	FSCN1	196.1
A0A2G8KǪX2	Putative ras-related protein	RAP1A	216.1
A0A2G8KP67	Gelsolin	GSN	178.7
A0A2G8KMT7	Non-muscle actin II	ACTB	744.2
A0A2G8KMD3	Putative ras-related protein	RAB1A	325.5
A0A2G8KHF6	Putative filamin-B	FLNC	463.8
A0A2G8K9B6	Putative ras-related protein	RAB2A	371.7
A0A2G8K865	Tropomyosin	TPM3	186.0
A0A2G8K252	Putative ras-related protein	RAB2A	368.2
A0A2G8K0R0	Tubulin alpha chain	RAB2A	371.7
A0A2G8JV23	Putative myosin-2 essential light chain-like isoform X2	MYL1	145.2
A0A2G8JǪV5	Alpha-tubulin	TUBA1A	297.0
A0A2G8JǪG7	Putative membrane-associated progesterone receptor component 1	PGRMC1	103.2
A0A2G8JPT8	Putative ras-related protein	RAB10	266.5
A0A2G8JE62	Putative filamin-B	FLNB	785.4

Cluster B (**Figure 3**) is linked to BP terms such as pentose-phosphate shunt, nucleosome assembly, response to oxidative stress, and carbohydrate metabolism. MF terms were linked to different functions of the pentose-phosphate pathway, such as transketolase activity; cellular oxidant detoxification, such as glutathione peroxidase activity; and the carbohydrate metabolic pathway, such as alpha-amylase activity (**Figure 5**). The KEGG analysis was in accordance with the GO findings previously shown (**Figure 5**). A detailed list of proteins linked to the STRING analysis is in the **Supplementary Material** and a graphical representation of the main pathways in which proteins of cluster B are involved is shown in **Figure 4**. (**Supplementary Material, Table**, sheet GO and KEGG cluster B, sheet proteins B). A STRING homology search was also applied for all the proteins belonging to cluster B to evidence the sequence homology between *H. tubulosa* and *H. sapiens*. The results of STRING homology search are shown in **Table 3**. The STRING similarity scores were between 750.0 (HAL), the highest homology score, and 95.5 (GPX3). Most of the molecules showed a similarity score of approximately 200.

**Table 3:** STRING homology analysis of cluster B proteins.

Protein ID	Protein name	Human homolog (Gene ID)	STRING similarity bit score
A0A2G8LPV2	Transaldolase	TALDO1	398.3
A0A2G8LPI7	3-hydroxyisobutyrate dehydrogenase	HIBADH	426.8
A0A2G8LNP3	Putative voltage-dependent anion-selective channel protein 2-like	VDAC2	409.8
A0A2G8LH72	Putative ELAV-like protein 3 isoform X2	ELAV3	370.5
A0A2G8LDJ7	Alkaline phosphatase	ALPI	424.9
A0A2G8LD14	Pancreatic alpha-amylase	AMY2B	292.0
A0A2G8L3H0	Pseudouridine-5’- phosphatase	PUDP	218.0
A0A2G8KVL2	nucleosome assembly protein (NAP) family	SET	315.8
A0A2G8KUJ2	Transketolase-like protein 2	TKT	242.7
A0A2G8KT35	Histone H2B	H2BU1	192.6
A0A2G8KT32	Histone H2B	H2BU1	194.5
A0A2G8KMI3	Calreticulin	CALR	542.0
A0A2G8KJV3	Glutathione peroxidase	GPX3	156.0
A0A2G8KDA9	Histidine ammonia-lyase	HAL	750.0
A0A2G8JZ09	Glutathione peroxidase	GPX3	95.5
A0A2G8JV41	Putative N- acetylgalactosamine kinase	GALK2	467.6
A0A2G8JH77	Fructose-bisphosphate aldolase	ALDOA	382.5
A0A2G8JH41	Alpha-amylase	AMY2B	396.4

The proteins of cluster C (**Figures 3**, **4**) can be grouped into three sub-clusters: proteins linked to primary cilium and BBsome, proteins linked to UPS machinery, and proteins related to ribosomal machinery. The BP terms of this cluster were as follows: cytoplasmic translational elongation, axonemal dynein complex assembly, positive regulation of the proteasomal ubiquitin-dependent protein complex, sperm axoneme assembly, and non-motile cilium assembly. MF terms associated with cluster C are large subunit rRNA binding and structural constituents of the ribosome. Finally, the CC terms of cluster C are BBsome, cytosolic large ribosomal subunit, ciliary membrane, non-motile cilium, and centrosome (**Figure 5**). Furthermore, the KEGG analysis was in accordance with the GO results previously shown (**Figure 5**). The **Supplementary Material** (**Supplementary Material Table**, sheet GO and KEGG cluster C, sheet proteins C) provides a detailed list of the proteins linked to the STRING analysis. A STRING homology search was also applied for all the proteins belonging to cluster C to evidence the sequence homology between *H.tubulosa* and *H. sapiens*. The results of the STRING homology search are shown in **Table 4**. Most molecules showed a similarity score higher than 200. Finally, some of the proteins belonging to all three clusters of intracellular fractions, such as Tubulin-A, Rab, F-actin capping protein, Cdc42, and Polyubiquitin were linked to both the Wnt and Hedgehog pathways. In particular, RhoA, and Dynein were present exclusively in the Wnt pathway, and Calreticulin, Tubulin-beta, BBS, and Elav were present in the Hedgehog pathway.

**Table 4:** STRING homology analysis of cluster C proteins.

Protein ID	Protein name	Human homolog (Gene ID)	STRING similarity bitscore
A0A2G8LR50	Ribosomal-like protein	RPL12	270.0
A0A2G8LLF3	Putative Bardet-Biedl syndrome 7 protein	BBS7	829.7
A0A2G8L1M0	NDK domain-containing protein	NME5	78.6
A0A2G8KUX1	Putative ribosomal protein P2	RPLP2	150.2
A0A2G8KCS4	Putative tetratricopeptide repeat protein 12	TTC12	286.2
A0A2G8KC39	Putative polyubiquitin-B isoform X2	UBC	271.6
A0A2G8K8H1	Phosphatidylethanolamine-binding protein	PEBP1	170.2
A0A2G8K524	Putative tetratricopeptide repeat protein 12	TTC12	296.2
A0A2G8JZP1	Putative 60S ribosomal protein L23a	RPL23A	201.4
A0A2G8JRJ1	Putative ribosomal protein P2	RPLP2	105.1
A0A2G8JCI2	Putative Bardet-Biedl syndrome 7 protein-like isoform X1	BBS7	946.0
A0A2G8JCA0	Ribosomal-like protein	RPL12	175.6

## 4 Discussion

Invertebrates rely solely on innate immunity, which includes both humoral and cellular responses, as they lack an adaptive immune system. Various methods to counteract infectious agents include coagulation, cell agglutination, encapsulation, and phagocytosis (1, 2). The microbial load in the natural marine habitat can number up to 10^6^ bacteria per mL and 10^9^ viruses per mL of seawater. Therefore, animals have developed a sophisticated innate immune system for survival.

**Figure 6:**
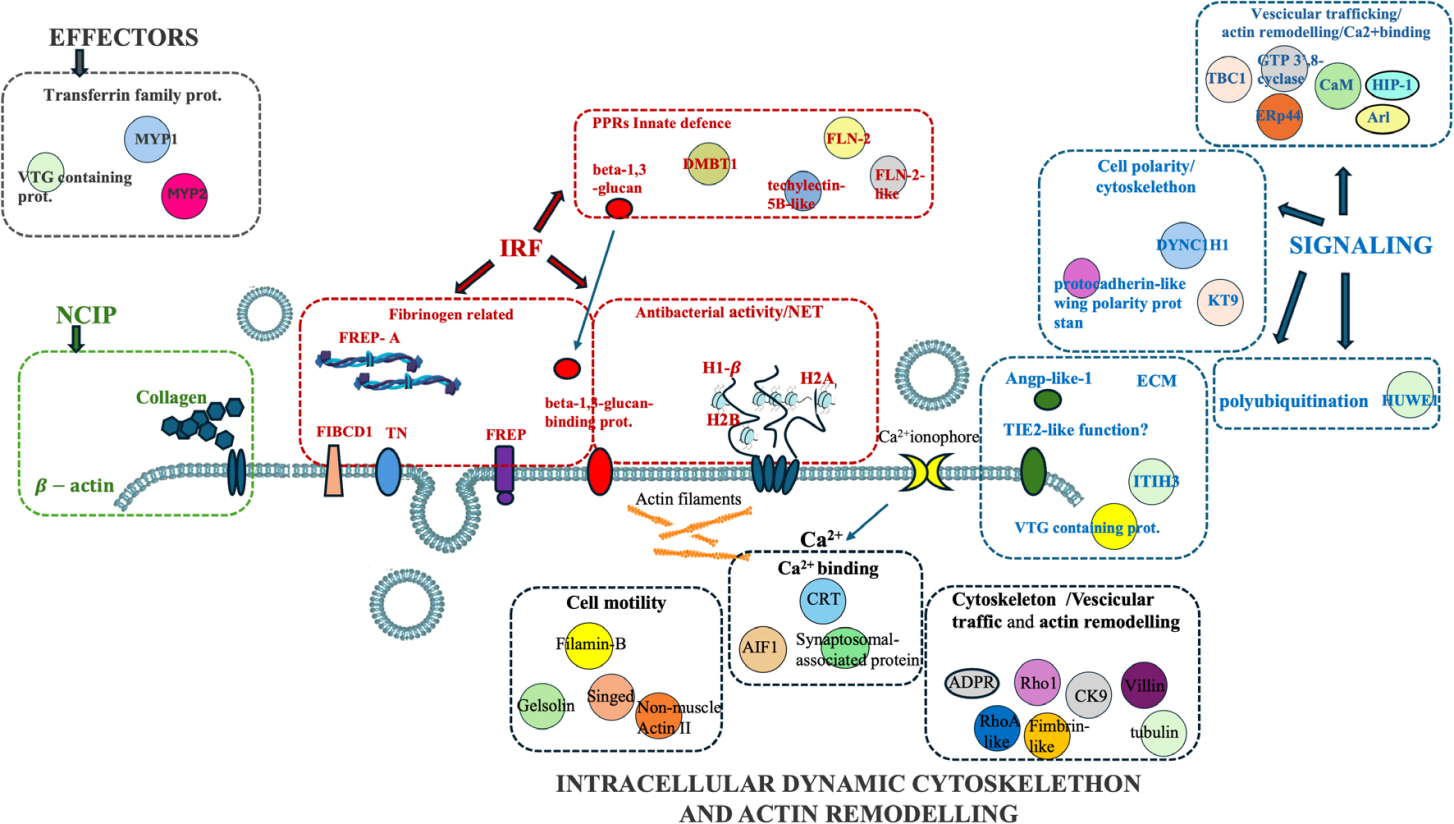
Graphical summary of extracellular and intracellular *H. tubulosa* proteins found in coelomic fluid.

In this study, proteomics analysis evidenced the presence of different groups of proteins in the extracellular and intracellular protein fractions of the coelomic fluid of the *H. tubulosa* organism (summarized in **Figure 6**). In particular, four classes of immune-related proteins have been evidenced in the humoral component (**Table 1**) and they are the following: IRFs, NCIPs, signaling molecules, and effector proteins. Moreover, the intracellular protein fraction evidenced the presence of different proteins linked to cytoskeleton, actin remodeling, different metabolic pathways, and finally ribosomal activity, ubiquitinilatyon, and primary cilium (**Tables 2**–**4**).

To differentiate between the self and the non-self, the invertebrates produce a series of PRRs, which can recognize molecular patterns associated with pathogens (PAMPs) (37). PRRs recognize PAMPs located on the surface of invasive cells, to subsequently neutralize and/or eliminate these invaders by various pathways. Currently, some receptors in echinoderms are known, including toll-like receptors (TLRs), NOD-like receptors (NLRs), lectins, and FREPs (38–41).

In invertebrates, FREPs have been identified in several species, including sponges, mollusks, horseshoe crabs, and ascidian (42–45). In invertebrates, these FREPs share a C-terminus with high sequence similarity to that of the fibrinogen (FBG) domain in humans, but they differ in the N-terminal portion.

In the extracellular protein fraction of *H. tubulosa* different IRFs have been identified and between them, there are the Ficolins. In particular, the proteomic analysis found the putative ficolin2 and Ficolin2-like. They belong to FREP family proteins with key roles in the lectin pathway. The ficolin- or MBL (mannose-binding lectin)-MASP (MBL-associated serine protease) complex binds directly to carbohydrates present on the surface of a variety of Gram-negative or Gram-positive bacteria, and subsequently, the complex initiates the lectin pathway to active the complement system, increasing the expression of the complement components, such as C3b, and initiating the lysis of bacteria through the membrane attack complex (46, 47). In general, Ficolins recognize the sugars present on microorganisms and enhance phagocytosis (47).

Proteomic analysis evidenced other proteins classified as IRFs, such as Techilectin 5B-like and DMBT1. They seem to intervene in host defense in different species tissues that serve as environmental barriers, such as the gut or the lungs of *H. sapiens*. Moreover, DMBT1 is upregulated in response to inflammation (48).

In the *H. tubulosa* humoral fraction, collagen was identified and linked with both host infection and structural ECM changes activated during pathogen infection (49).

In the gastropod *Biomphalaria glabrata*, a proteomic analysis evidenced different classes of immune-related genes classified as IRFs, such as the C-type and H-type lectins, and NCIPs, such as collagen (50).

Moreover, other non-structural components of the ECM identified are the tenascins (TNs), defined as “Matricellular proteins,” and only expressed during specific tissue conditions in inflammation (51). These include angiopoietin-like -1, putative angiopoietin-2-like, putative inter-alpha-trypsin inhibitor heavy chain H3 (ITIH3), and VTG-containing protein. Despite their classical role in the ECM, there is evidence of the immunological functions of these latter proteins. For instance, Sun et al. (52) showed that distinct Vitellogenin domains in the Chinese mitten crab (*Eriocheir sinensis*) showed definitive bacterial binding activity *via* interaction with the signature components on microbial surfaces, and that this function may be conserved between different species due to the conserved amino acid residues. All these proteins were also evidenced in the sea cucumber species *Apostichopus japonicus* (26).

Other molecules identified in the *H. tubulosa* proteome are histones H1-β, H2A, and H2B.

In addition to their canonical role in guiding chromatin structure folding and regulating the transcriptional process, histones contribute to innate immune responses as antimicrobial peptides. Several mechanisms regulating their role as antimicrobial agents against host pathogens have been described in vertebrates (53–56) and invertebrate species. Between these roles, extracellular traps (ETs) and histones in lipid droplets are produced and accumulate, which can be selectively released in response to immune stimuli (40, 41).

The first evidence of NETosis in invertebrates (called ETs) was shown in *Galleria mellonella* honeycomb moths and *Litopenaeus vannamei* shrimp by observing the participation of extracellular nucleic acids in the immune system (57). Some globular domain structures with smooth fibers associated with ETs were observed in these invertebrates, and they were later identified as histones (58, 59). After these reports, ET formation in invertebrates was studied, suggesting their role in the invertebrate immune response.

The intracellular protein fraction of coelomatic fluid of *H. tubulosa* evidenced proteins linked to actin remodeling, cytoskeleton, cell signaling, different metabolic pathways, ribosomal activity, UPS machinery, and primary cilium.

In particular, some proteins in Cluster A have a putative role in cellular mechanisms linked to host infection response, such as villin, gelsonin, and allograft inflammatory factor-1 (AIF-1). In humans, villin proteins are involved in an actin comet formed by intracellular bacteria (60). Gelsonin, a protein involved in cell motility, has a role in the modulation of the host antimicrobial response (61). In invertebrates, AIF-1, a protein linked to calcium-binding activity, may play an important role not only in immune responses to alloantigens but also in various host responses to inflammatory stimuli (62). In *H. tubulosa*, the presence of these protein classes allows us to hypothesize that putative vesicular trafficking and cytoskeleton remodeling guide some cellular mechanisms linked to cell polarity.

In cluster B, the intracellular protein fraction identified different proteins linked to metabolic pathways. In particular, pancreatic alpha-amylase, involved in polysaccharide hydrolysis in smaller subunits (63), was shown in Holothuroidea (64), indicating a digestive maturity in these animals. Moreover, glutathione peroxidase was identified. It is involved in glutathione metabolism, which converts glutathione disulfide (GSSG), the oxidized state of glutathione, into a reduced form (GSH). These are also oxidative stress markers in *H. tubulosa* (65). Other proteins belonging to the pentose-phosphate pathway, such as transaldolase (TA) and transketolase (TK), were present. Tricarboxylic acid cycle (TCA) proteins found in MS data were 3-hydroxybutyrate dehydrogenase. These metabolic pathways have fundamental functions in cellular metabolism, such as maintaining carbon homeostasis, providing precursors for nucleotide and amino acid biosynthesis, and reducing oxidative stress (66). Nagy (67) showed that in vertebrates’ immune system, some cellular types intervening in first defense mechanisms, such as neutrophils or macrophages, can reconfigure these metabolic pathways, defined as “switching to the cyclic pentose phosphate pathway powers”.

Finally, in cluster C, proteins linked to ribosomal activity, UPS machinery, and primary cilium were evidenced.

*H. tubulosa* protein data also show a BBsome structure (BBS-7-like and BBS-7-like-X1 isoform), an essential regulator of the ciliary protein composition (68).

As previously reported, some of the proteins belonging to all three clusters of the intracellular fraction, such as Tubulin-A, Rab, F-actin capping protein, Cdc42, and Polyubiquitin were linked to both the Wnt and Hedgehog pathways. In particular, RhoA and Dynein were present exclusively in the Wnt pathway, and calreticulin, Tubulin-beta, BBS, and Elav were present in the Hedgehog pathway.

Moreover, among the humoral proteins of the coelomic fluid, we evidenced the presence of fibrocystin, a protein that in humans is hypothesized to play regulatory roles in primary cilia-mechanosensation, calcium signaling, and planar cell polarity (69).

**Figure 7:**
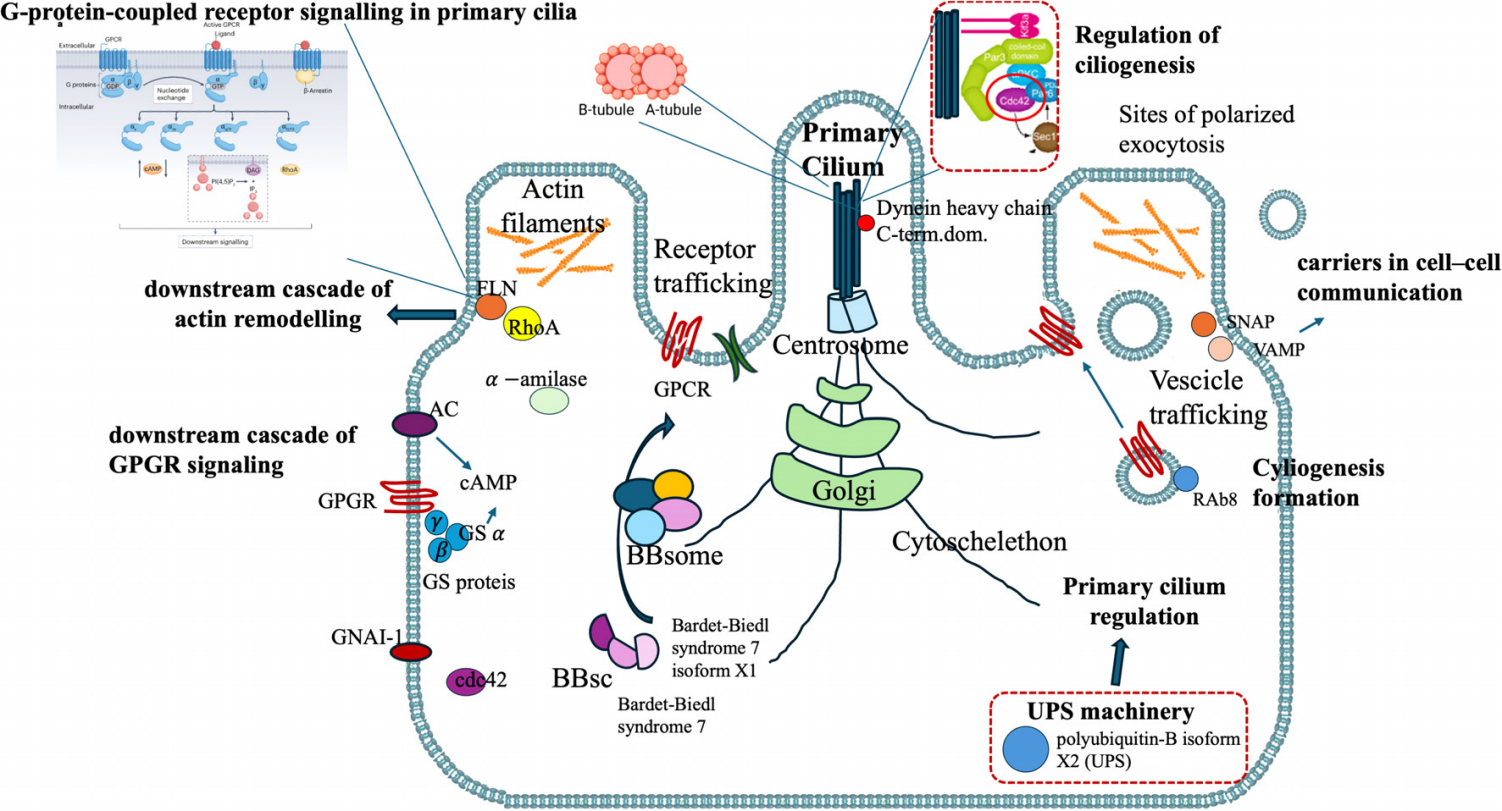
*H. tubulosa* intracellular protein fraction linked to a putative structure resembling primary cilium.

In humans, the primary cilium is considered a signaling hub for a multitude of molecules, linked to different cellular pathways linked to homeostasis, including the Hedgehog, Wnt, Notch, Hippo, GPCR, PDGF (and other RTKs including FGF), mTOR, and TGF-beta pathways, that allow the cell to respond to various external stimuli (70). The presence of proteins linked to primary cilia (**Figure 7**) in the intracellular component of the *H. tubulosa* proteome, together with the presence of proteins involved in pathways such as Hedgehog and Wnt, suggest that in *H. tubulosa*, there could also be a putative primary cilium structure involved in cell signaling in homeostasis. This study aims to cover knowledge gaps regarding the mechanisms modulating specific immune cell phenotypes, behaviors, and functions highly conserved through evolution.

## Data Availability

The original contributions presented in the study are included in the article/supplementary material (extracellular and intracellular fraction spectra sheets), by mean of the protein spectra analyzed through Mass Spectrometry and processed for downstream data analysis; further inquiries on raw data can be directed to the corresponding author/s.
